#  Protective effects of 2-methoxycinnamaldehyde an active ingredients of *Cinnamomum cassia* on warm hepatic ischemia reperfusion injury in rat model

**DOI:** 10.22038/IJBMS.2019.13987

**Published:** 2019-12

**Authors:** Hannaneh Golshahi, Atefeh Araghi, Farshad Baghban, Saeed Farzad- Mohajeri

**Affiliations:** 1 Nanobiotechnology Research Center, Avicenna Research Institute, ACECR, Tehran, Iran; 2 Department of Clinical Sciences, Faculty of Veterinary Medicine, Amol University of Special Modern Technologies, Amol, Iran; 3 Department of Veterinary Medicine, Yasooj Branch, Islamic Azad University, Yasooj, Iran; 4 Department of Surgery and Radiology, Faculty of Veterinary Medicine, University of Tehran, Tehran, Iran; 5 Institute of Biomedical Research, University of Tehran, Tehran, Iran

**Keywords:** Ischemia reperfusion injury, Liver, Oxidative stress, Rat 2-Methoxycinnamaldehyde

## Abstract

**Objective(s):**

Hepatic ischemia/reperfusion injury (IRI) is one of the major causes of hepatic failure during liver transplantation, trauma, and infections. The present study investigated the protective effect of intra-portal administration of 2-methoxycinnamaldehyde (2-MCA) on hepatic IRI in rats.

**Materials and Methods:**

Twenty-four rats were equally divided into four groups; 1) sham group, (no IRI or transfusion), 2) Hepatic IRI (60 min ischemia + 120 min reperfusion, 3) Hepatic IRI+ NS (IRI + normal saline), 4) Hepatic IRI+2-MCA, (IRI + 2-MCA). In groups 3 and 4, 1 ml/kg normal saline and 2-MCA were administered slowly into the vein of the left lateral and median lobes of the liver 10 min before induction of hepatic reperfusion (upper the site of clumping), respectively. The harvest time points were at 2 hours post-reperfusion in all groups.

**Results:**

Histologically, cell death, degenerative changes, sinusoidal dilatation, congestion, hemorrhage, and infiltration of inflammatory cells were observed in IRI group, while these pathological changes were attenuated in the 2-MCA administrated group. The level of alanine transaminase, aspartate transaminase, tumor necrosis factor- α and interleukin-6 in serum and hepatic malondialdehyde were significantly increased by IRI, and 2-MCA administration reduced all these markers. In addition, caspase-3 and nuclear factor κB (NF-κB) expression were investigated immunohistochemically. Administration of 2-MCA considerably decreased caspase-3 positive cells and NF-κB activity in comparison with IRI group.

**Conclusion:**

As a conclusion, in situ administration of 2-MCA protects against hepatic IRI via anti-inflammatory, and anti-apoptotic properties.

## Introduction

Ischemia reperfusion injury (IRI) is a well-defined phenomenon during cellular damage in organs, which is caused by hypoxia, and is paradoxically exaggerated after the restoration of oxygen delivery (1). The warm IRI that is initiated by hepatocellular damage develops during various liver surgery (e.g. transplantation), trauma, shock, and toxic liver injuries and may lead to liver and/ or multi-organ failure (2, 3). Pathophysiology of hepatic IRI is associated with several factors like: the interaction among hepatocytes, liver sinusoidal and endothelial cells (LSEC), Kupffer cells (KCs), hepatic stellate cells (HSCs), infiltration of inflammatory cells, activation of platelets, generation of reactive oxygen species (ROS) and reactive nitrogen species (RNS), increased levels of adhesion molecules, and release of cytokines and chemokines (4-6).

 The mitochondrial permeability transition (MPT) plays an important role in the pathogenesis of tissue injury after IRI (5, 7). Permeability transition pores non-specifically cause conduction of low weight molecules up to 1500 Da and make mitochondrial depolarization, uncoupling of oxidative phosphorylation, and high amplitude mitochondrial swelling. ATP depletion after uncoupling leads to oncotic necrosis, while swelling causes outer membrane rupture, release of pro-apoptotic factors, like cytochrome C and caspase activation (6, 7).

The nuclear factor κB (NF-κB) as a transcription factor plays an important role in the regulation of a large number of genes that are involved in the physiological processes, including cell survival, cell death, inflammation, and immune responses. The activated NF-κB regulates the expression of different genes encoding inflammation-related transcripts such as acute phase proteins, cell adhesion molecules, cell surface receptors, cytokines and chemokines, as well as anti-apoptotic and pro-apoptotic proteins (8, 9). Matsui *et al. *(2000) reported that both lipid peroxidation and NF-κB activation occur during the warm ischemic period (10). To determine the role of NF-κB in IRI of the rat liver, its activity was analyzed by electrophoretic mobility shift assay (EMSA). Findings have shown that NF-κB is activated within 1 hr and 2 hr after the initiation of reperfusion and decreased after 4 hr. Also, mRNA expression of tumor necrosis factor- α (TNF-α) and intercellular adhesion molecule 1 (ICAM-1) genes are increased 2 hr after the reperfusion. So, it can be concluded that during hepatic IRI, NF-κB is activated and can bind to a specific sequence in the promoters of budget genes, which can up-regulate the expression of TNF-α and ICAM-1 mRNA resulting in IRI in the liver of the rats (11).

The genus *Cinnamomum* comprises over 250 aromatic evergreen trees distributed mostly in Asia (12). The bark and twigs of *Cinnamomum cassia* is being used for alleviation of common cold, cough, diabetes, fever, flatulence, indigestion, sinusitis, sore throat and as a general tonic tea for a varied range of symptoms including digestive disorders, blood purification, immunostimulation and as an antiparasitic compound (13-15). Cinnamaldehyde (CIN) naturally exists in various species of the genus *Cinnamomum *and is used in preparing beverages, medical products, perfumes, and cosmetics. CIN possesses antioxidant, antibacterial, antifungal, anti-viral, anti-neoplastic, anti-mutagenic, anti-inflammatory, anti-hyperglycemic, anti-hyperlipidemic, peripheral vasodilator, and cytotoxic properties (16-18).

Recently, it has been demonstrated that 2-methoxycinnamaldehyde (2-MCA), one of the active ingredients of the cortex of the *C. cassia, *has antioxidant, anti-inflammatory, anti-proliferative, immunomodulatory, and anti-diabetic properties (19, 20). Trans-cinnamaldehyde and 2-MCA were identified as NF-κB inhibitors from *C. cassia *with IC50 values of 43 μM and 31 μM, respectively. Both compounds inhibited lipopolysaccharide (LPS)–induced DNA binding activity of NF-κB in addition to NF-κB transcriptional activity (21). Considering these beneficial properties, here we assessed the protective effect of intra-portal administration of 2-MCA on hepatic IRI in rats.

## Materials and Methods

***Animals***

A week before initiation of experiments, 24 male Wistar rats (*Rattus norvegicus*), aging 8 weeks and weighing 180-200 g were purchased from Pasteur Institute of Iran (Tehran, Iran). Animals were maintained in caging system, under standard condition such as room temperature (22±2 ºC), relative humidity (50-60%), and a 12 hr light – dark cycle and were allowed access to commercial standard rat pellet and tap water *ad libitum*. All animals received human care in compliance with the Guide for Care and Use of Laboratory Animals published by the National Institutes of Health (NIH publication No. 85-23, revised 1985). The animal protocol was planned to minimize pain and discomfort to the animals, and every effort was made to minimize animal suffering. 

***Preparation of 2-MCA***

We purchased 2-MCA from Sigma Aldrich as powder (Product Number: 289019). For preparation of injection solution, it was dissolved in 0.05% Tween-20 using double distilled water.

***Study design***

Rats were randomly divided into the following four groups with six rats in each group: Group 1: (Sham), the liver was exposed after laparotomy, and no occlusion of the vessels of the liver was performed. Group 2: (IRI), the animals were subjected to partial (70%) hepatic warm ischemia model for 60 min and reperfusion induced for 120 min after ischemia. In group 3 (IRI+ normal saline) and group 4 (IRI+ 2-MCA) 1 ml/kg normal saline (NS) and 1 ml/kg 2-MCA were injected slowly into the vein of the left lateral and median lobes of the liver 10 min before hepatic reperfusion, respectively. Further procedures were the same as those of group 2. 

***Experimental model***

The animals were anesthetized via intra-peritoneal injection of ketamine (75 mg/kg) and xylazine (7.5 mg/kg). Under sterile condition, an identical midline abdominal incision was performed. The animals were kept supine for the duration of the experiments. Throughout all procedures, rectal temperature was maintained between 36-37°C by the heating pad. Partial warm hepatic IRI was performed by *Pringle Maneuver*. Briefly, after detaching of liver from its ligaments, the portal vein, hepatic artery, and biliary branch of left lateral and middle hepatic lobes were occluded by a traumatic microvascular clamp (22). This method induced ischemia approximately in 70% of liver, so the right lateral and caudate lobes had taken whole portal and arterial blood supply. This partial hepatic IRI can avoid splanchnic congestion and any confusing effects resulting from bowel ischemia or hemodynamic disturbances (23). Diminishing the color of the liver tissue was used as a positive marker for confirmation of ischemia. During clamping, the liver was replaced into the abdominal cavity and the incision was closed temporarily to decrease evaporative loss. Reperfusion was confirmed by an immediate back to normal color. In the groups with intra-portal administration, solutions were slowly injected by a 32 gage needle into the vein of the left lateral and median lobes of the liver (upper the site of clumping) 10 min before finishing of ischemic period. After that, the clamps were removed and reperfusion occurred (22).

***Sample collection***

For collecting samples, after reperfusion for 120 min, 2 ml blood from right ventricle of the heart of each animal were harvested and centrifuged at 3000 rpm for 15 min at 4 ^°^C. The separated serum stored in -70 ^°^C for next complementary tests. After euthanasia, samples from hepatic median and left lateral lobes were collected, fixed in 10% phosphate-buffered formalin solution and also, immediately frozen in liquid nitrogen, and then stored at -80 ^°^C for further evaluation. For histopathological analysis, ten accidental parts of each slide were examined under ×20 objective. Severity of the damages was semi-quantitatively evaluated. The assessed histopathological parameters were: congestion, swelling, vacuolar degeneration, oncotic necrosis, apoptotic necrosis, leukocyte infiltration, and tissue hemorrhage. These pathologic changes were scored as follows: 0, no alteration; 1+, less than 10%; 2+, less than 50%; 3+, more than 50% of the cells are affected. The maximal score attainable by any individual sample was 21. 

***Immunohistochemical staining***

4-µm thickness sections were heated to 60 ^°^C for 30 min, deparaffinized and hydrated in three changes of xylene and serial alcohol. Then, the slides were washed in Tris-buffered saline (TBS). Endogenous peroxidase activity was quenched by incubating the slides in 3% hydrogen peroxide at 37°C for 10 min. A rabbit polyclonal antibody against active caspase-3 (Cat#ab13847, Abcam) (diluted 1:100) and NF-κB (Cat ab#16502, Abcam) (diluted 1:500) were used and slides were incubated at 4 ^°^C overnight and then incubated with secondary antibody, goat anti-rabbit IgG (diluted1:100) 1 hour at room temperature (RT) in the dark. After washing with TBS, slides were incubated with diaminobenzidine tetrahydrochloride (DAB) as the substrate, and counterstained with hematoxylin. Negative control slides without primary antibodies were used in the same manner. Positive reaction for caspase-3 and NF-κB reactivity was detected by evaluation of 5 randomly selected areas at a magnification of ×400. The cleaved caspase-3 positive rate was expressed as the ratio of brown cells to blue cells. The positive reaction of NF-κB in each photograph was evaluated and the average of all photographs in each group was calculated.

***Biochemical analysis***

*Measuring the level of transaminases*

The serum levels of alanine transaminase (ALT) and aspartate transaminase (AST) were measured by standard commercial biochemical assay kits, using Olympus AU400 Chemistry Analyzer (Manufactured by Diagnostic Systems Group of Olympus America Inc. USA). The levels were expressed as unit per liter. All samples were tested in duplicate.

*Determination the level of cytokines in serum*

Serum levels of TNF-α and interleukin-6 (IL-6) were determined using an Elisa kit (Rat IL-6 Elisa Kit Bioscience, Cat No: SK00110-02; Rat TNF-alpha Elisa Kit Bioscience, Cat No: SK00109-02, respectively) according to the instructions of the manufacturer. Concentrations were expressed in pictogram per milliliter. All samples were tested in duplicate.

*Determination the level of hepatic malondialdehyde*

Hepatic malondialdehyde (MDA) levels were measured by the thiobarbituric acid reaction according to the method of Buege and Aust (1978) (24). At first, frozen liver tissue was homogenized and boiled in a solution containing glacial acetic acid, thiobarbituric acid, and NaCl buffer. After cooling to room temperature, the mixture was centrifuged at 1500 rpm for 15 min. MDA reacts with thiobarbituric acid forming a solution pink in color. MDA was measured in the supernatant by spectrophotometry (UV752, Shanghai, China) at a wavelength of 532 nm using a method described by Ohkawa *et al* (25). MDA concentrations were expressed as nanomoles per mg of protein.

***Statistical analysis***

Statistical analyses and evaluations were performed using Windows-compatible SPSS 20.0 software. The distribution of groups was analyzed using the *Kolmogorov Smirnov* test. The normality test showed that the data were consistent with a normal distribution. The data were analyzed by One-way ANOVA (Analysis of Variance) and *post-hoc* Tukey HSD (Honestly Significant Difference) test. For analyses of histopathological changes, the Kruskal-Wallis variance was used and the group medians were compared by Mann-Whitney U (Bonferroni) test when differences between them were detected. All values were expressed as mean±standard deviation (SD) and *P*< 0.05 was considered statistically significant.

## Results

**Figure 1:**
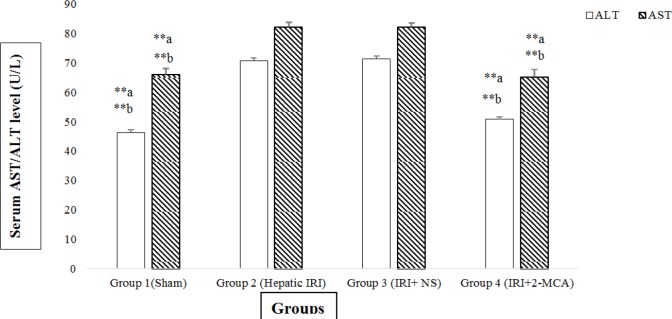
Effect of intra-portal administration of 2-MCA on the level of AST and ALT in IRI model. **P<0.001.a versus group 2, b versus group 3. All the values were expressed as mean±SEM. 2-MCA: 2-methoxycinnamaldehyde, ALT: Alanine aminotransferase; AST: Aspartate aminotransferase; IRI: Ischemic-reperfusion injury

Hepatic ischemia for 60 min and reperfusion for 120 min resulted in a significant increase in the serum levels of ALT and AST compared to sham- operated group (*P*<0.001 and *P*<0.001, respectively. However in group received 2-MCA, the average amounts of ALT was significantly lower compared to those of IRI group and normal saline-received group, respectively (*P*<0.001 and *P*<0.001, respectively). Similar reduction was detected in the level of AST in the 2-MCA-received group compared to the IRI and IRI+ NS groups (*P*<0.001 and *P*<0.001, respectively)(Figure 1).

**Figure 2:**
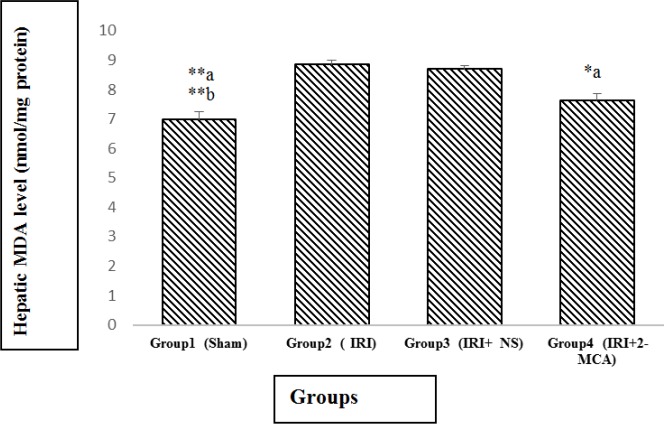
Effect of intra-portal administration of 2-MCA on the level of hepatic MDA in IRI model. *P<0.05, **P<0.001.a versus group 2, b versus group 3. All the values were expressed as mean±SEM. MDA: Malondialdehyde, 2-MCA: 2-methoxycinnamaldehyde, IRI: Ischemic-reperfusion injury

The average amounts of MDA in the IRI group and IRI+ NS was markedly higher in comparison with sham group (*P*<0.001 and* P*<0.001, respectively). There was significant decline in MDA in IRI+2-MCA group in comparison with IRI group (*P*<0.05) (Figure 2).

**Figure 3:**
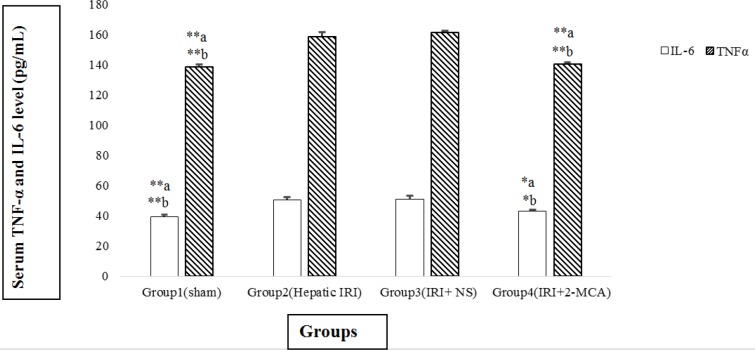
Effect of intra-portal administration of 2-MCA on the level of TNF-α and IL-6 in IRI model. *P<0.05, **P<0.001.a versus group 2, b versus group 3. All the values were expressed as mean±SEM. TNF-α: Tumor necrosis factor-alpha, IL-6: Interleukin-6, IRI: Ischemic-reperfusion injury, 2-MCA: 2-methoxycinnamaldehyde

The content of IL-6 in IRI and IRI+ NS groups was dramatically higher than the sham group (*P*<0.001 and *P*<0.001, respectively). Similar results were detected in the TNF-α level. The average amount of IL-6 in IRI + 2-MCA showed statistically decreasing in comparison with IRI and IRI+NS groups (*P*<0.05 and* P*<0.05, respectively) (Figure 3). Additionally, significant decline in the level of TNF-α was observed in group received 2-MCA when compared to IRI and IRI+ NS groups (*P*<0.001 and *P*<0.001, respectively) (Figure 3). 

**Figure 4:**
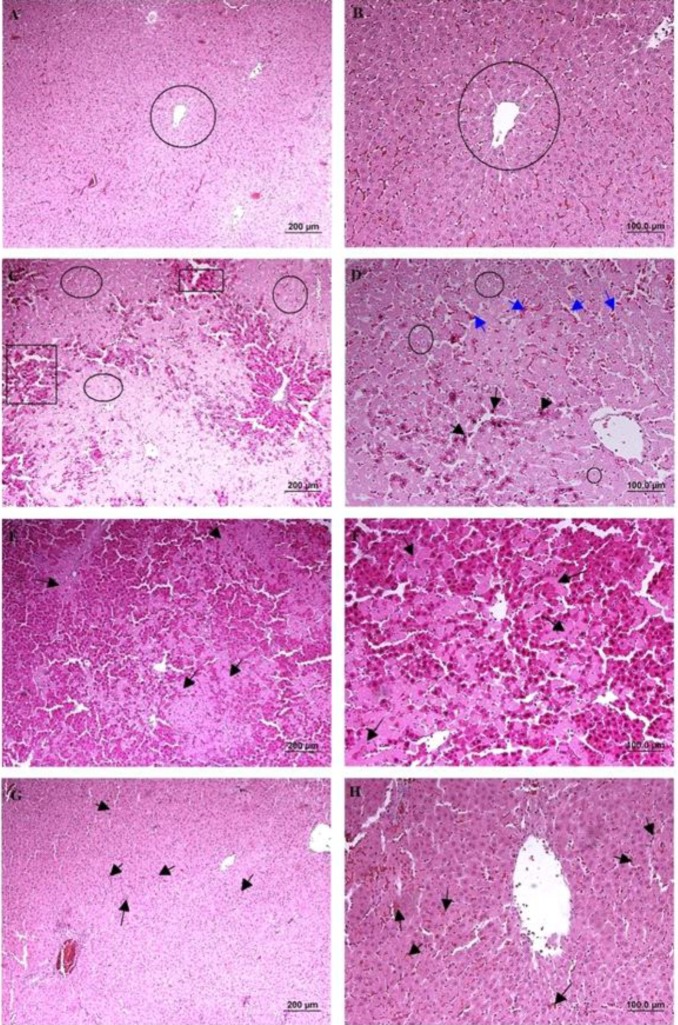
Male Wistar rats were subject to partial warm hepatic IRI with intra-portal administration of NS and 2-MCA at dose of 1 ml/kg 10 min before finishing of ischemic period. Liver damage was assessed 2 hr after reperfusion (A & B):Sham-operated rat, cords of hepatocytes are arranged radially around the central vein (circle), (C): Rat undergoing 1 hr of ischemia followed by 2 hr of reperfusion, note to serve injury characterized by necrotic hepatocytes (rectangle) and degenerative changes (circle), (D): Higher magnification of previous Figure, note to hydropic degeneration (circle), pyknotic nuclei (black arrow), and congestion (blue arrow), (E & F): (IRI+ NS), Note to karyolysis in the nuclei of hepatocytes (arrow), (G & H): (IRI+ 2-MCA), Noticeable restoration of hepatocyte integrity and slight congestion (arrow) were detected (H& E, scale bar: A, C, E, and G: 200 μm , B, D, F, and H: 100 μm). IRI: Ischemic-reperfusion injury; 2-MCA: 2-methoxycinnamaldehyde; NS: Normal saline

According to the microscopical examination, no pathologic alterations were observed in the group 1 (sham group) (Figure 4A and B). In the group 2 (hepatic IRI), 1 hr of ischemia and 2 hr of reperfusion induced significant injuries and lobular distortion. Cell death (apoptotic necrosis and oncotic necrosis) was the main histopathologic lesion, which was occurred with focal and/or random distribution pattern. In zone I and II, the most prominent features were vacuolar change and hydropic degeneration of hepatocytes. Infiltration of inflammatory cells (especially polymorphonuclear cells) was observed in areas of hepatic injury. Dilation of sinusoidal space, vascular congestion and some foci of hemorrhage were also noted (Figure 4C and D). In group 3 (IRI + NS), extensive vacuolar changes, hydropic degeneration, necrotic and also apoptotic cells were detected with relatively the same intensity with group 2. Moderate neutrophilic infiltration in parenchyma and also portal area and severe congestion were also noted (Figure 4E and F). In group 4 (IRI + 2-MCA), hepatocyte damage was ameliorated significantly compared to previous groups, and apoptotic and necrotic features were significantly decreased. Only hydropic degeneration and mild congestion were detected. Sinusoid expansion was much decreased compared to the group 2 and 3 (Figure 4G and H). The IRI group had a significantly higher score than the sham group (*P*<0.01), and intra-portal administration of 2-MCA significantly ameliorated the score of injury (*P*<0.01) (Table 1).

**Figure 5:**
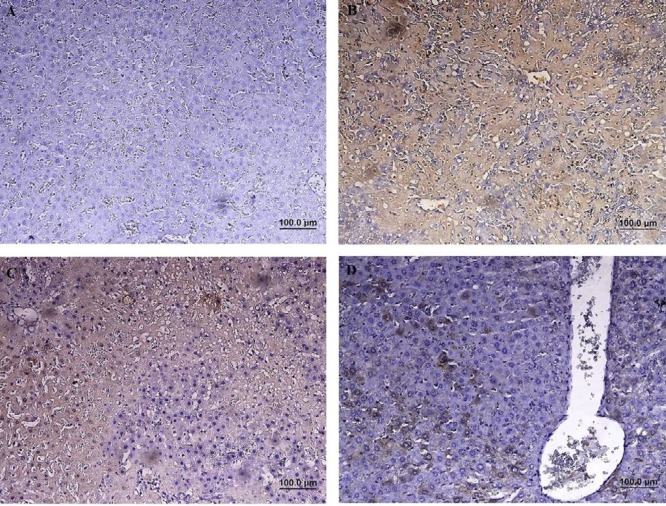
Immunohistochemical staining of cleaved caspase-3 in hepatic tissue, (A):Sham-operated rats showing no expression of cleaved caspase-3, (B) IRI demonstrating significant caspase-3 activity, (C): (IRI+ NS), Note to prominent immunopositivity in cytoplasm of hepatocytes, (D): (IRI+ 2-MCA), scatter expression of caspase-3 (IHC, scale bar: 100 μm). IRI: Ischemic-reperfusion injury; 2-MCA: 2-methoxycinnamaldehyde; NS: Normal saline

**Figure 6:**
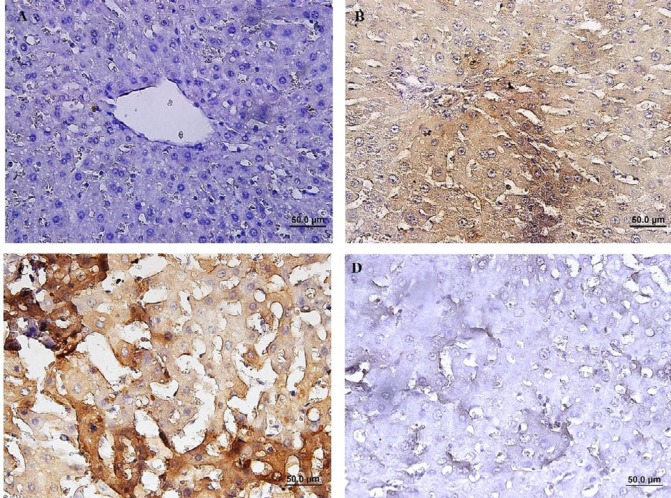
Immunohistochemical staining of NF-kB in rat liver, (A): Sham-operated rats showing no expression of NF-KB. (B): IRI showing a significant increase in NF-kB immunoreactivity in the cytoplasm of hepatocytes. (C): (IRI+ NS), Brown color indicates NF-kB positivity, (D): (IRI+ 2-MCA), demonstrating a significant reduction in NF-kB immunostaining (IHC, scale bar: 100 μm). IRI: Ischemic-reperfusion injury, 2-MCA: 2-methoxycinnamaldehyde, NS: Normal saline, NF-kB: Nuclear factor-kB

Immunohistochemical (IHC) staining of caspase-3 revealed that the number of cleaved caspase-3- positive cells in group with IRI was significantly increased compared to the sham-operated group (*P*<0.01). The percentage of cleaved caspase-3-positive cells was decreased in group received 2-MCA compared to IRI and NS administrated groups (*P*<0.01) (Figure 5). Activated NF-κB was assessed by immunocytochemistry and showed many positively stained cells in IRI group. However, administration of 2-MCA 10 min before initiation of reperfusion period could significantly decrease the NF-κB activity (Figure 6).

**Table 1:** Comparison of the effect of intra-portal administration of 2-MCA on microscopic damage in rats exposed to hepatic IRI

**Group**	**Total injury score**
**Group 1 (Sham)**	0.0 (0.0-1.0)
**Group 2 (Hepatic IRI)**	18.0 (14.0-18.0) ^a^
**Group 3 (IRI+ NS)**	16.0 (13.0-16.0)^a^
**Group 4 (IRI+2-MCA)**	4.0 (2.0-9.0)^ab^

Values are median (minimum-maximum)

a Significantly increased in groups 2-3 compared to that in group 1 (*P*<0.01)

b Significantly decreased in groups 4 compared to that in group 2 and 3 (*P*<0.01, *P*<0.01, *P*<0.01, respectively). IRI: Ischemic-reperfusion injury; 2-MCA: 2-methoxycinnamaldehyde; NS: Normal saline

## Discussion

Hepatic IRI occurs substantially during liver resection or transplantation and remains a major cause of liver failure following liver surgery. The factors/pathways have been associated with the hepatic IRI process include oxidative stress, anaerobic metabolism, inflammatory cells, mitochondria, intracellular calcium overload, KCs, cytokines, and chemokines (26). In the early stages of reperfusion phase, swelling of endothelial cell, vasoconstriction, and presence of inflammatory cells result in failure of the microcirculation, so, large areas of the liver remain ischemic after the onset of reperfusion. The second phase is mostly the result of production of inflammatory cytokines and oxygen-derived free radicals (27). Therefore, an effective method for preventing or minimizing hepatic IRI during liver surgery is desired. To develop a new approach to decrease liver IRI, the present study focused on the protective effects of intra-portal administration of 2-MCA before starting of the reperfusion period on liver in rat model.

For evaluation of liver function, the level of ALT and AST was determined. The results indicated that the levels of aforementioned enzymes were increased by IRI, whereas 2-MCA treatment could significantly decrease the level of hepatic enzymes suggesting that it could attenuate hepatic IRI. Tabatabaei *et al.* (2015) showed that pre-administration of cinnamon extract in diets of broiler chickens inoculated with *Escherichia coli* could meaningfully reduce the gene expression levels of pro-inflammatory mediators and liver enzymes activities (28). In another study, the elevated serum AST and ALT enzymatic activities induced by carbon tetrachloride (CCl_4_) were considerably restored to normal level by oral administration of 200 mg/kg of aqueous and ethanolic extracts of cinnamon once daily for 7 days, as compared to control group (29).

 Oxidative stress plays a vital role in IRI. ROS mainly acts on proteins, enzymes, nucleic acids, cytoskeleton, and lipid peroxides, leading to mitochondrial dysfunction and lipid peroxidation. ROS can also harm endothelial cells and destroy the integrity of the microvasculature (26). ROS have the ability to oxidize polyunsaturated fatty acids of hepatocyte membranes and cause lipid peroxidation that leads to alteration in cell membranes fluidity, inactivation of some of membrane-bound enzyme, and increasing membrane permeability finally leading to cell death. MDA is a marker of peroxidation injury induced by ROS (30). In this study, the level of MDA in IRI and IRI + normal saline groups increased significantly in comparison with sham group, whereas the level of MDA decreased noticeability by 2-MCA. Some previous works revealed that administration of cinnamaldehyde could decrease oxidative stress level, lipid abnormalities and inflammatory markers in the liver and the muscles of a fructose-fed rat (31, 32). The extract of *C. cassia* has been reported to have hepatoprotective property probably due to its free radical scavenging activity. The effect of alcoholic extract of cinnamon bark in a mouse model of acute alcohol-induced steatosis showed that pretreatment with cinnamon extract significantly reduced the hepatic lipid accumulation. Also, the effect of ethanol extract from *C. cassia Blume* (CCE) on the activation of hepatic stellate cells (HSCs) significantly reduced the expression of alpha-smooth muscle actin (a-SMA), connective tissue growth factor (CTGF), transforming growth factor beta1 (TGF-b1) and tissue inhibitor of metalloproteinase-1(TIMP-1) (33). Cinnamaldehyde with anti-oxidative and anti-inflammatory properties also diminished the ischemic myocardial injury in rats (34). *Cinnamomum cassia* has been reported to have anti-inflammatory activity through the potent inhibition of nitric oxide (NO) and cyclooxygenase (35). Other researchers reported that cinnamaldehyde alleviated gestational hyperglycemia in rats through modulation of peroxisome proliferator-activated receptor gamma (PPARγ), pro-inflammatory cytokines and oxidative stress (36). It is known that ROS do not cause cytotoxicity directly, but act as signaling molecules that up-regulate NF-κB and subsequently release TNF-α and IL-1. Activated KCs significantly increase the releasing of ROS and pro-inflammatory cytokines, including TNF- α, IL-1, IL-6, IL-8, and IL-12. Both TNF- α and IL-1 up-regulate Mac-1 (CD11b/CD18) adhesion proteins on neutrophils and induce IL-8 synthesis, further promoting neutrophil chemotaxis within the parenchyma. Moreover, IL-1 has the potential to stimulate the release of ROS by neutrophils, which will further increase TNF- α synthesis by KCs (37, 38). TNF- α also induces P-selectin expression in liver sinusoidal endothelial cells, which is essential for the recruitment of neutrophils (39). TNF- α has been shown to increase the release of other molecules, including IL-6, macrophage inflammatory protein-2 (MIP-2), epithelial neutrophil activating protein-78 (ENA-78), cytokine-induced neutrophil chemoattractant- 1 (CINC), and a number of CXC motif chemokines (including CXL-1, -2, and -3). In addition, IL-1 and TNF- α recruit and activate CD4+ T-lymphocytes, which produce granulocyte-macrophage colony- stimulating factor (GM-CSF), interferon gamma (INF-γ), and TNF-β. These cytokines amplify KC activation and encourage neutrophil recruitment and adherence into the liver sinusoids (40-42). This study indicated that 2-MCA as an anti-inflammatory agent could decrease the amount of pro-inflammatory cytokines and contributed to reduction of damages induced by IRI. Zhao *et al.* (2015) demonstrated that 2-MCA reduced brain edema and brain injuries through reduction of expression of receptors and factors such as toll-like receptor 4 (TLR4) and NF-κB as well as reduction of inflammatory mediators such as TNF- α and IL-1β (43). 2-MCA has been shown to have an inhibitory effect on LPS-induced NF-κB transcriptional activity (21). Cinnamaldehyde could inhibit NF-κB, p-IκB and IκB protein and mRNA abundance in dorsal root ganglion neurons, which were associated with decreased release of pro-inflammatory cytokines (TNF-a and IL-6) and increased neuronal survival under high glucose (HG) conditions (44), and all of these findings were in accordance with our results.

Administration of 1 ml/kg 2-MCA, 10 min before reperfusion period decreased NF-κB activity significantly. Activated NF-κB is translocated to the nucleus and acts as a transcription factor that promotes the expression of genes involved in regulation of the inflammation and immune gene encoding cytokines, pro-inflammatory enzymes and adhesion molecules, pro-apoptotic and anti-apoptotic molecules of cell cycle, or cellular invasion. In previous studies, the expression of NF-κB in hepatic tissue and nuclear translocation of activated NF-κB, and hepatic content of TNF-α in CCl4-treated group were significantly increased, while in rats received cinnamaldehyde, the expression of NF-κB and the level of TNF-α was meaningfully reduced (45-47). Zakaria *et al.* (2016) indicated that cinnamaldehyde could suppress TLR4 signaling pathway and reduced TNF-α level, which are involved in NF-κB activation via the canonical pathway; however, they assumed that cinnamaldehyde might have further suppressed NF-κB activation by non-canonical pathway, which can be triggered, independently of TNF-α, by B-cell activation factor (BAFFR), lymphotoxin β-receptor (LTβR), CD40 and receptor activator for NF-κB (RANK)(45). Researchers also found that the inhibitory effects of trans-cinnamaldehyde and 2-MCA on NF-κB activation were not attributed to their antioxidant properties since synthetic derivatives of cinnamaldehyde and cinnamic acid such as a-methyl cinnamaldehyde and caffeic acid did not show significant antioxidant activity and inhibitory properties on NF-κB transcriptional activity even at 100 μM (21). Furthermore, trans-cinnamaldehyde and 2-MCA as the NF-κB inhibitors revealed dose-dependent inhibitory effects on LPS and/or zymosan (48).

The release of chemical mediators during IRI resulted in increased mitochondrial membrane permeability, which cause activating and releasing of many proteins involved in apoptosis, such as caspases, and cytochrome C. This sequence of events leads to DNA destruction and onset of apoptotic necrosis process (49). In addition, release of inflammatory mediators during IRI causes both apoptotic and oncotic necrosis (50).

It has been shown that 2-hydroxycinnamaldehyde modulates the pro- and anti-apoptotic proteins (caspase-3 and Bcl-2) (51). Lee *et al.* (2006) stated that the extract of cinnamon inhibits the proliferation of HT-29 colon cancer cells, but not CCD-112CoN normal colon cells (52). The finding of other research indicated that Aqueous Cinnamon Extract (AEC) inhibited tumor cell growth (in HeLa, Caco-2, EL4, and Clone M3 cell lines), but not in primary normal mouse lymphocyte (53). Use of 75 mg/kg of *Cinnamomum zeylanicum *for 4 weeks in rats, as an antioxidant in food, increased the level of superoxide dismutase (SOD), glutathione peroxidase (GPX), and catalase (CAT) that leads to the elimination of ROS and lipoperoxidation (LPO) and also the apoptotic index (54). Cinnamaldehyde reduced the HG-induced expression of NF-κB, IκBα and p-IκBα, TNF-α and IL-6. Furthermore, it significantly suppressed the overexpression of cleaved caspase-3 induced by HG. These findings indicate that cinnamaldehyde has a neuroprotective role in HG-induced injury in a dose-dependent manner (44). 

## Conclusion

To conclude, a Alleviation in hepatic injuries in group treated with 2-MCA could be attributed to anti-inflammatory and anti-oxidative, and anti-apoptotic effects.
